# Spectroscopic Studies of *R*(+)-α-Lipoic Acid—Cyclodextrin Complexes

**DOI:** 10.3390/ijms151120469

**Published:** 2014-11-07

**Authors:** Naoko Ikuta, Akira Tanaka, Ayako Otsubo, Noriko Ogawa, Hiromitsu Yamamoto, Tomoyuki Mizukami, Shoji Arai, Masayuki Okuno, Keiji Terao, Seiichi Matsugo

**Affiliations:** 1 Graduate School of Medicine, Kobe University, Kobe 650-0017, Japan; E-Mails: naoko.ikuta@people.kobe-u.ac.jp (N.I.); keiji.terao@cyclochem.com (K.T.); 2 College of Science and Engineering, Kanazawa University, Kanazawa 920-1192, Japan; E-Mails: akira.tanaka.621@gmail.com (A.T.); amm21843@gmail.com (A.O.); peridot@staff.kanazawa-u.ac.jp (T.M.); ultrasa@staff.kanazawa-u.ac.jp (S.A.); mokuno@staff.kanazawa-u.ac.jp (M.O.); 3 Department of Pharmaceutical Engineering, School of Pharmacy, Aichi Gakuin University, Nagoya 464-8650, Japan; E-Mails: noriko30@dpc.agu.ac.jp (N.O.); hiromitu@dpc.agu.ac.jp (H.Y.); 4 CycloChem Bio Co., Ltd., Kobe 650-0047, Japan

**Keywords:** cyclodextrin, lipoic acid, microscopic FT-IR, microscopic Raman, raman spectroscopic mapping

## Abstract

α-Lipoic acid (ALA) has a chiral center at the C6 position, and exists as two enantiomers, *R*(+)-ALA (RALA) and *S*(−)-ALA (SALA). RALA is naturally occurring, and is a cofactor for mitochondrial enzymes, therefore playing a major role in energy metabolism. However, RALA cannot be used for pharmaceuticals or nutraceuticals because it readily polymerizes via a 1,2-dithiolane ring-opening when exposed to light or heat. So, it is highly desired to find out the method to stabilize RALA. The purpose of this study is to provide the spectroscopic information of stabilized RALA and SALA through complexation with cyclodextrins (CDs), α-CD, β-CD and γ-CD and to examine the physical characteristics of the resultant complexes in the solid state. The RALA-CD structures were elucidated based on the micro fourier transform infrared (FT-IR) and Raman analyses. The FT-IR results showed that the C=O stretching vibration of RALA appeared at 1717 cm^−1^ and then shifted on formation of the RALA-CD complexes. The Raman spectra showed that the S–S and C–S stretching vibrations for RALA at 511 cm^−1^ (S–S), 631 cm^−1^ (C–S) and 675 cm^−1^ (C–S) drastically weakened and almost disappeared upon complexation with CDs. Several peaks indicative of O–H vibrations also shifted or changed in intensity. These results indicate that RALA and CDs form host-guest complexes by interacting with one another.

## 1. Introduction

**Figure 1:**
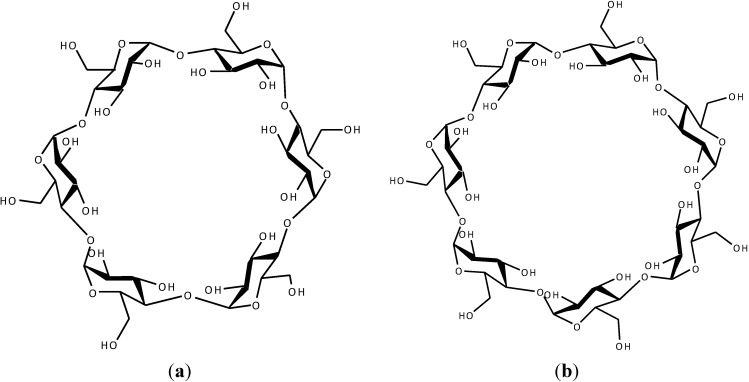

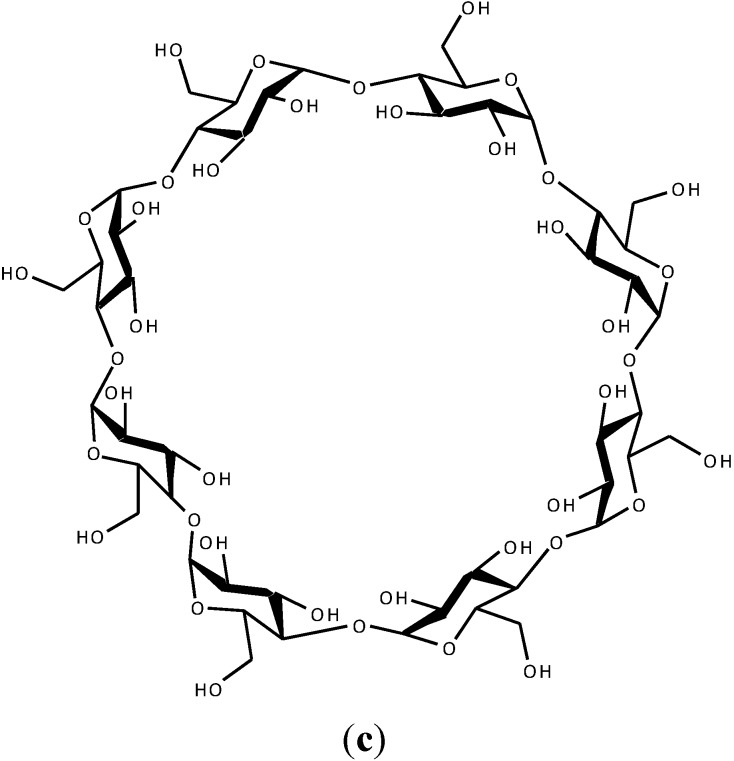
Chemical structures of (**a**) α-cyclodextrin (α-CD); (**b**) β-cyclodextrin (β-CD); and (**c**) γ-cyclodextrin (γ-CD).

Cyclodextrins (CDs) are cyclic oligosaccharides, comprised of six (α-CD), seven (β-CD) or eight (γ-CD) α-1,4-linked glycopyranose units. They have hydrophilic hydroxyl groups on the outer surface and a hydrophobic cavity at the center (Figure 1). As a result, CDs are capable of forming complexes with a variety of ionic and lipophilic substances by taking the entire molecule or part of it into the cavity and can affect the aqueous solubility, stability or bioavailability of the guest [1,2,3,4]. The molecular recognition phenomena by CDs have therefore been a subject of special attention in the past decades as suitable models for enzyme-substrate binding process and/or receptor-drug interactions in which the various functionalized groups play an important role [5,6,7]. The structure of such a complex has been of great interest, and many have tried to explain it using binding constants or by considering the ring size of the CD [8,9].

**Figure 2:**
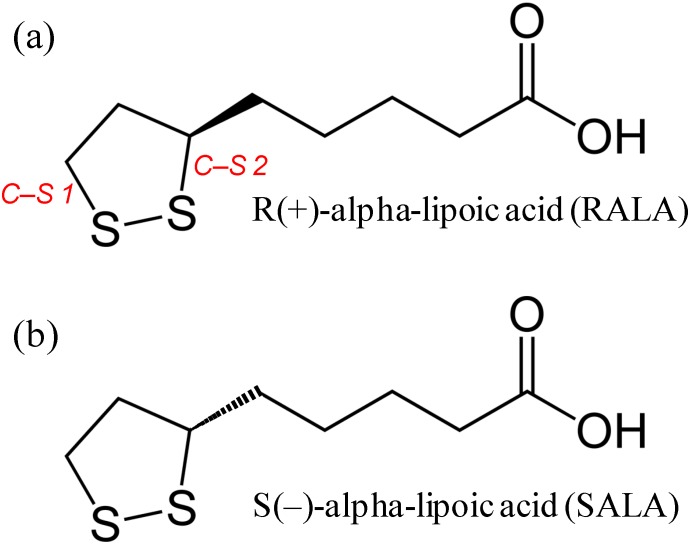
Chemical structures of (**a**) *R*(+)-ALA (RALA); and (**b**) *S*(−)-ALA (SALA). (*C-S1*:C8-S bond. *C-S2*:C6-S bond.)

These experiments were mostly carried out in the liquid phase, and peak shifts or new peak appears or some peak disappears upon complexation were observed with NMR and UV-VIS spectroscopy. Unfortunately, these changes could only be measured when the guest molecule interacted strongly with a CD, so molecules with weak binding constants have not yet been thoroughly studied. Many CDs and their derivatives are now commercially available, and various experimental techniques to accurately measure them in the solid state have also been developed (e.g., differential scanning calorimetry (DSC), scanning electron microscopy (SEM), X-ray diffraction (XRD)). Analysis of the interaction between a host and guest with a weak binding constant requires an alternative analytical technique. α-Lipoic acid (ALA) is a strong antioxidant, and has both a hydrophobic and a hydrophilic part (Figure 2). ALA has a partition coefficient of approximately 4/1 (*o*/*w*) [10], meaning that ALA, unlike other antioxidants, is soluble in both aqueous and non-aqueous media. ALA has a relatively weak affinity for CDs [11], so it is difficult to accurately analyze the spectral changes using UV or FT-IR spectroscopy. Microscopic FT-IR can now be used to give an new analytical method that any other published analytical data of ALA-CD complexes [12,13,14]. ALA has one S–S bond and two C–S bonds in its 1,2-dithiolane and also has a chiral center at the C6 carbon, leading to two enantiomers, *R*(+)-ALA (RALA) and *S*(–)-ALA (SALA) (Figure 2), of which RALA is the naturally occurring compound. The beneficial effects of RALA are reported, that it regulates the cell’s redox status, increases glucose uptake through recruitment of the glucose transporter-4 to plasma membranes, improves glucose disposal in patients with type II diabetes, and can work as a regulator of energy expenditure in mice [15,16,17]. Therefore RALA can provide potential use as nutraceuticals, pharmaceuticals and cosmeceuticals. The commercially available ALA is the racemate and many researchers used racemate [4,11,12,13,14,18,19,20] and the study featured RALA is not enough. RALA is the only bioactive form but is unstable when exposed to low pH, light or heat [21]. We have recently shown that it is possible to stabilize RALA through complex formation with γ-CD yielding RALA-CD. In the previous work we investigated the physicochemical properties of RALA-CD complexes using DSC, SEM, XRD and HPLC analysis; however, the interaction between the host and guest was not investigated in the solid state [22,23]. In this study, aiming to provide the spectroscopic information of RALA-CD and SALA-CD complexes, we initially tried to evaluate the interaction of RALA and SALA with CDs using FT-IR, but it is quite difficult to observe the vibration of the S–S or the C–S bond in 1,2-dithiolane of RALA and SALA in FT-IR spectroscopy because of the poor intensity. The state of S–S bond relates to the stability of ALA. The focus then turned to Raman spectroscopy to investigate the S–S or the C–S bond in 1,2-dithiolane of RALA and SALA complexed with CDs, which has not yet been studied. Herein, we report the formation of RALA-CD and SALA-CD complexes using Microscopic FT-IR, Microscopic-Raman and XRD. Furthermore, Raman Spectroscopic Mapping of the RALA-CD and SALA-CD complexes was used to compare their physical mixtures.

## 2. Results and Discussion

For the ALA-CD complexes and the physical mixtures investigated in this study, ALA and the corresponding molar amounts of the different CDs for a 1:1 ratio were prepared as we described before [22].

### 2.1. Microscopic Fourier Transform Infrared (FT-IR) Spectroscopy

IR spectroscopy is used to analyze CD complex formation because the bands for the guest molecule shift or change in intensity on complex formation [24]. In this study, the KBr pelleting method was used initially, however KBr readily absorbs moisture and the preparation of the thin film by mixing CD-complex with KBr changed the complex formation completely, so the direct measuring method, the microscopic IR methodology, was then adopted. The Nicolet iN 10 MX Infrared Imaging Microscope (Thermo Fisher Scientific, Waltham, MA, USA) was used to measure samples in the solid state.

**Figure 3:**
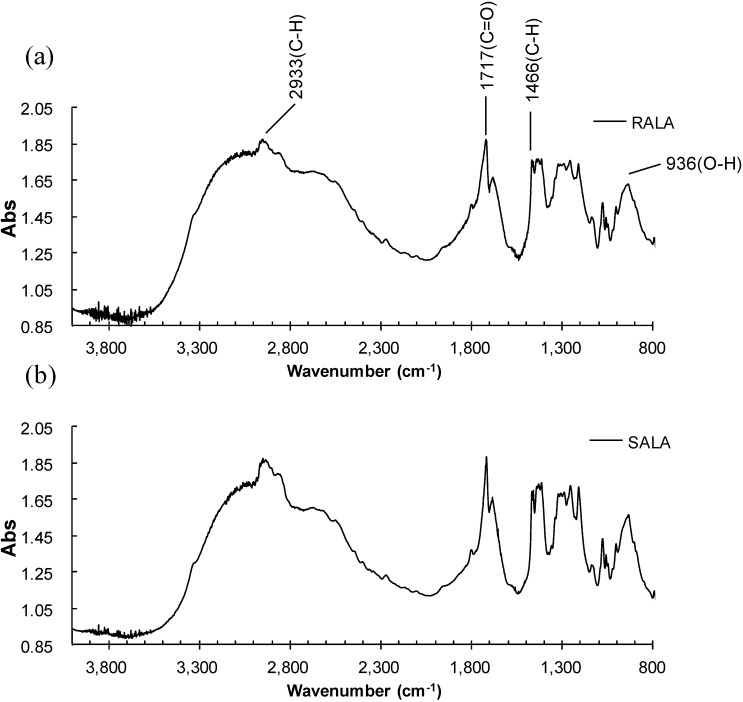
Microscopic Fourier transform infrared (FT-IR) spectra of (**a**) RALA and (**b**) SALA.

The IR spectra of RALA and SALA are shown in Figure 3 and the following characteristic bands were observed: 2933 (–CH_2_–); 1717 (C=O); 1466 (CH) and 936 (OH) cm^−1^. These bands are in accordance with the reported data [25], however the S–S and C–S bands were not observed.

**Figure 4:**
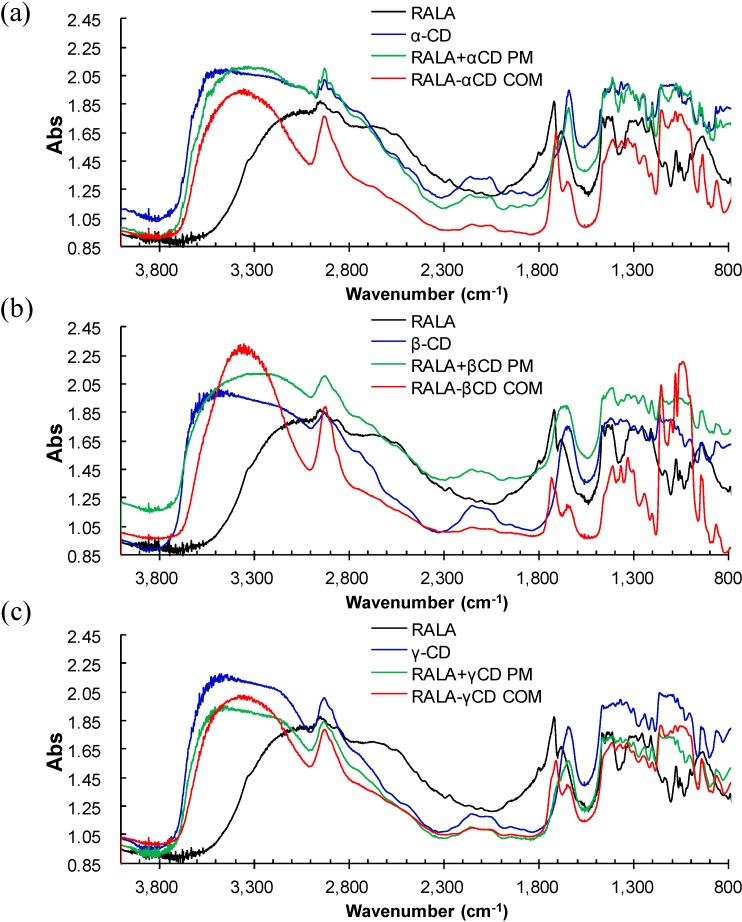
Micro-FT-IR spectra of RALA, RALA + CD physical mixture (PM) and RALA-CD complex (COM). ((**a**) α-CD; (**b**) β-CD and (**c**) γ-CD).

**Figure 5:**
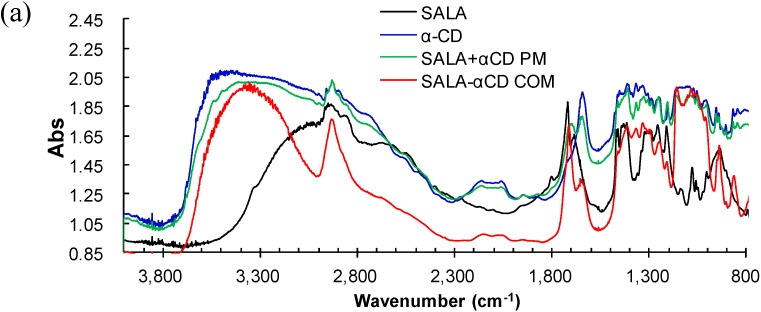

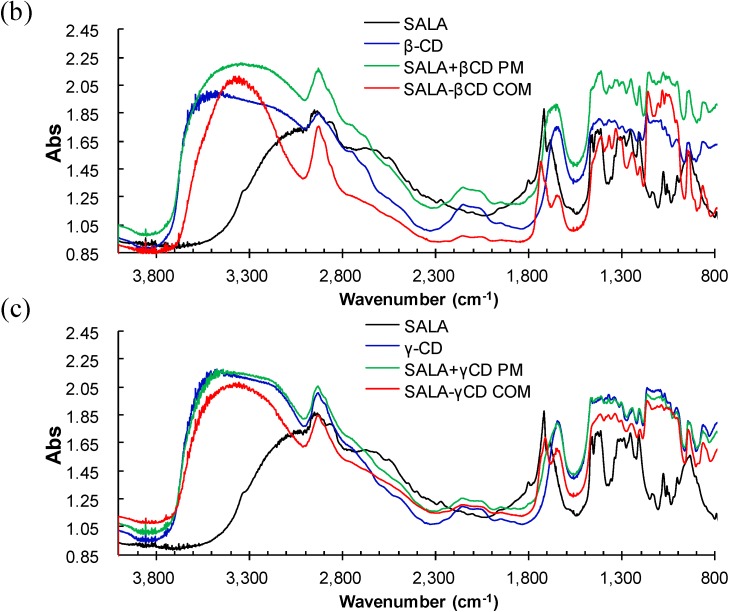
Micro-FT-IR spectra of SALA, SALA + CD physical mixture (PM) and SALA-CD complex (COM). ((**a**) α-CD; (**b**) β-CD and (**c**) γ-CD).

In the IR spectra of α-, β- and γ-CD (Figure 4 and Figure 5, blue) a wide band was observed with an absorption maximum at 3400 cm^−1^. This is due to the valence vibrations of either the O–H bonds of the primary hydroxyl groups (C6–OH) connected by intermolecular hydrogen bonding or the secondary hydroxyl groups connected by intramolecular hydrogen bonding (the C2–OH group of one glucopyranose unit and the C3–OH group of an adjacent glucopyranose) [26]. An absorption band is also observed, belonging to the valence vibrations of the C–H bonds in the CH and CH_2_ groups with a maximum at 2927 cm^−1^. The absorption bands from the deformation vibrations of the C–H bonds in the primary and secondary hydroxyl groups are observed in the region 1400–1200 cm^−1^. The bands from the valence vibrations of the C–O bonds in the ether and hydroxyl groups of the CDs are observed in the region 1200–1000 cm^−1^. The absorption bands in the region 950–800 cm^−1^ belong to the deformation vibrations of the C–H bonds and the pulsation vibrations of the glucopyranose cycle. The bands around the 1630 cm^−1^ region (Figure 4 and Figure 5), will reflect the δ-HOH bending of water molecules attached to CDs [27], the physical mixtures, and the complexes.

Upon RALA complexation with α-, β- or γ-CD the ν_as_ (C=O) shifted from 1717 cm^−1^ for RALA alone to 1709, 1733 or 1709 cm^−1^, respectively. The shift to a higher frequency can be attributed to the interruption of strong hydrogen bonding in RALA upon complex formation with β-CD. ALA has a relatively weak affinity for CDs and the reported values for the apparent binding constant of lipoate anion with the CDs are 158, 1644, and 70 M^−1^ for α-, β- and γ-CD, respectively [11]. Our result using the microscopic IR methodology showed a similar tendency with the reported binding constant. The ν_as_ (OH) (bending mode) located at 1640 and 1643 cm^−1^ in pure α-CD and β-CD, respectively appeared at 1635 and 1642 cm^−1^ upon complexation. However, no change was observed for complexation with γ-CD.

In the spectra for RALA-CD complexes the band at approximately 1700 cm^−1^ was assigned to the C=O stretching of RALA [13] and after complexation with β-CD this band shifted to a higher wave number, whereas for the physical mixture no shift was observed. This result suggests that there is interaction between RALA and β-CD in the solid complex, and carbonyl property of RALA-β-CD get stronger than that of free RALA. For α-CD and γ-CD, the C=O band shifted to a lower wave number after complexation.

**Figure 6:**
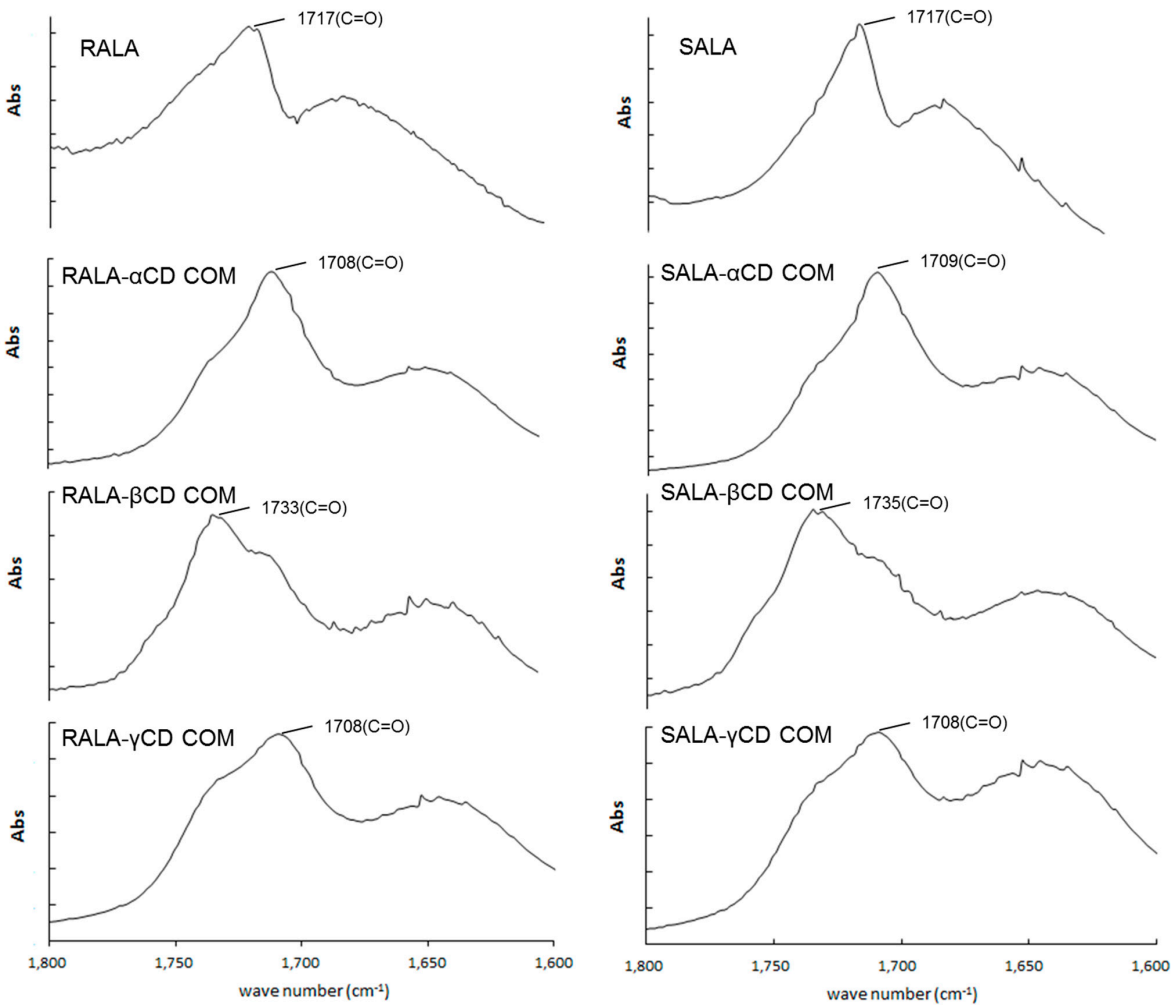
Enlarged micro-IR spectra of RALA-CD and SALA-CD complexes. (1600–1800 cm^−1^) (COM: Complex).

There was also no appearance of new bands in the FT-IR spectra of blends. The microscopic FT-IR detected the shoulder peak of the ν_as_ (C=O) band, which was not observed with the KBr method (data not shown), and it can be attributed to the coexistence of different association type species present in the solid state (Figure 6). The difference of the peak shifts direction between β-CD and α- or γ-CD results from the peak intensity ratio of the C=O bands around 1700 cm^−1^ , which analyzed with PeakFit v4.12 (Systat Software, Inc., San Jose, CA, USA) near 1710 and 1735 cm^−1^. This indicates that the carbonyl of the RALA-βCD complex exists in a more hydrophobic environment than that of RALA-αCD or RALA-γCD complexes. The same pattern was observed for SALA-CD complexes.

PeakFit v4.12 (Systat Software, Inc., San Jose, CA, USA) was used to automatically place peaks in the measured spectroscopy and find local maxima in a smoothed data.

These results indicate that C=O of lipoic acid can be included in β-CD cavity and forming complex, but for α- and γ-CD, the inclusion of C=O might not take place. Rácz *et al.* reported the crystal structure of the inclusion complex of β-CD with ALA (racemate) [20]. They showed a molecular packing of the inclusion complex of β-CD with racemate ALA, which found that the fatty acid chain of ALA was a bent conformation compared with the ALA model after geometry optimization in a vacuum [20]. Our data could provide new information for the RALA-βCD conformation. The S–S and C–S bands were not identified and will be confirmed with the results from Raman spectroscopy in the next chapter.

### 2.2. Microscopic Raman Spectroscopy and Raman Mapping Analysis

#### 2.2.1. Microscopic Raman Spectroscopy

**Figure 7:**
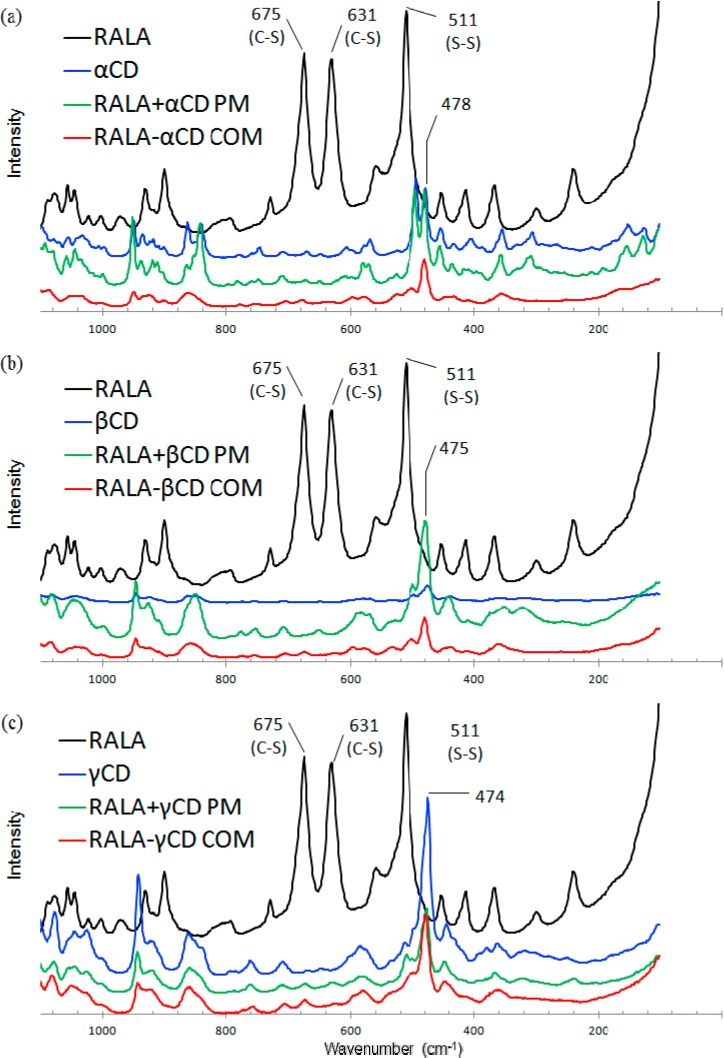
Raman spectra of RALA-CD complexes. ((**a**) α-CD; (**b**) β-CD and (**c**) γ-CD) (COM: Complex, PM: Physical mixture).

**Figure 8:**
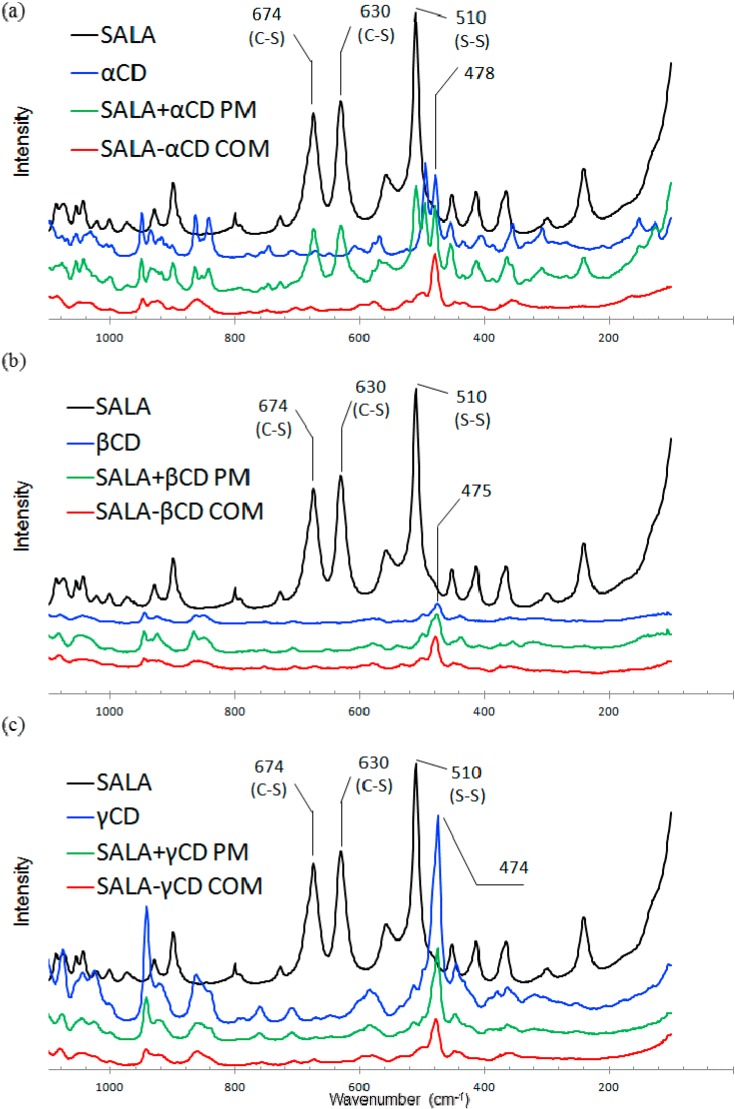
Raman spectra of SALA-CD complexes. ((**a**) α-CD; (**b**) β-CD and (**c**) γ-CD) (COM: Complex, PM: Physical mixture).

Resonance Raman spectroscopy can be used to analyze the effect of β-cyclodextrin complexation on the structural properties of several guest chemicals [28,29]. The aim of this study was to observe the 1,2-dithiolane of ALA using microscopic Raman spectroscopy. The Raman spectra are taken in the range of 80–1100 cm^−1^. The Raman spectra for RALA and SALA including their corresponding complexes and physical mixtures are shown in Figure 7 and Figure 8, respectively.

The ν_1_ band (S–S stretching) was observed around 510 cm^−1^ for RALA and SALA and the ν_2_ bands (C–S stretching) were observed around 630 and 675 cm^−1^ for RALA and SALA (Figure 7 and Figure 8, black). The higher wavenumber of ν_2_ was assigned to C–S 1 and the lower to C–S 2 (Figure 2) using density functional theory (DFT) and the Gaussian-09 program package [30]. Geometries were optimized at the B3LYP/6-311+(d,p) theory level and theoretical IR and Raman spectra of RALA were also calculated at the B3LYP/6-311+(d,p) theory level. The ν_1_ (S–S) and ν_2_ (C–S) mode for RALA + CD physical mixtures occasionally appeared depending on the place of the measurement for the test samples (see Figure 8a, green, appeared. The others not appeared). This was because the area irradiated by the laser was very quite limited and it is impossible to cover all area of RALA in the physical mixture. On the other hand, for these complexes, neither the ν_1_ nor ν_2_ mode was present. This suggests that localization of the S–S and C–S bonds in the RALA may change conformationally induced by complex formation with CD. These results indicated that the complexes were homogeneous, however the physical mixtures were heterogeneous. Therefore, it required an analysis covering a wide range and we conducted Raman spectroscopic imaging next.

#### 2.2.2. Raman Spectroscopic Imaging

**Figure 9:**
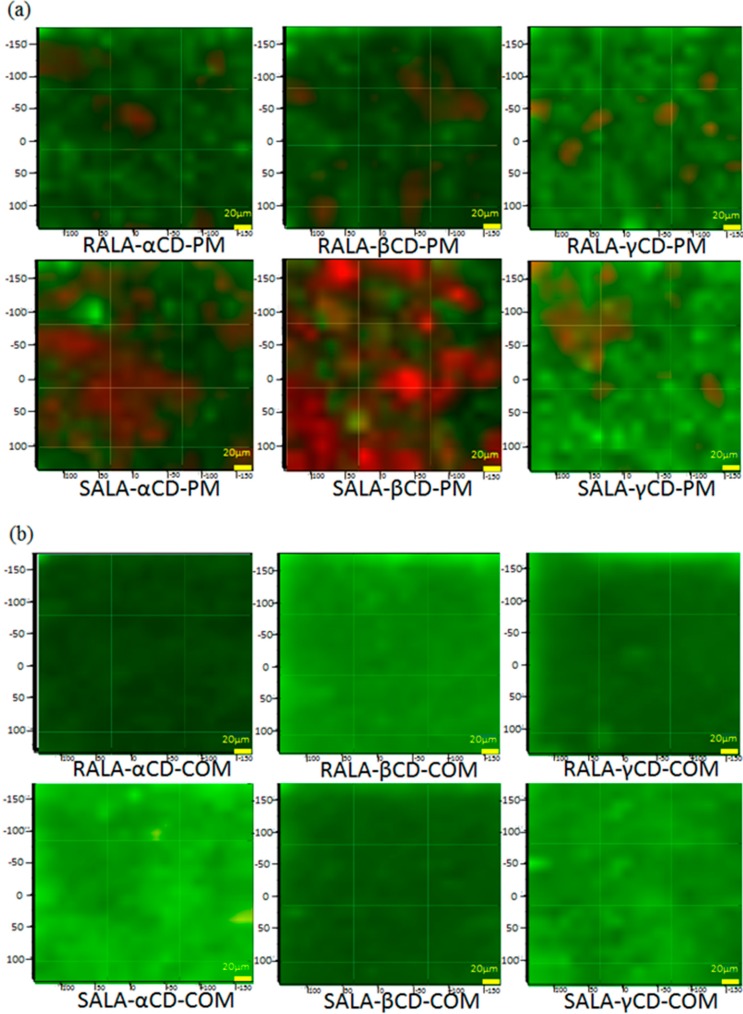
Raman chemical mapping of (**a**) the physical mixtures (PM) and (**b**) the complexes (COM). (Red: Lipoic acid, Green: CDs).

The Raman spectra for the physical mixtures showed occasional appearance of S–S and C–S peaks depending on the sample location. In these cases Raman chemical mapping was carried out on the RALA-CD and SALA-CD complexes and the equivalent physical mixture to confirm both the ν_1_ (S–S) and ν_2_ (C–S) mode (Figure 9). The visualized score images give insight into the spatial distribution and heterogeneity of the components in the physical mixtures, with RALA and SALA in red.

RALA and SALA are completely heterogeneous and present as distinct particles in the RALA + CD and SALA + CD physical mixtures; however, in the CD complexes there seem to be no RALA or SALA present in any pixel. The formation of a complex would result in identical spatial distribution of the two components and resulted in the disappearance of the S–S and C–S bands, indicating that in the physical mixtures no complex forms between the CDs and RALA or SALA. This is in accordance with the SEM particle size analysis we reported, which showed that the particle size distribution changed on complex formation, and with the XRD analysis, which showed the XRD pattern changed when a complex formed [22]. The XRD patterns were analyzed for SALA-CD complexes in this study and the pattern change occurred upon complex formation as for RALA-CDs [22] (data not shown).

It should be noted that a few points on the Raman score image prove the presence of non-complexed active lipoic acid (Figure 9b SALA-α-CD COM). According to Figure 7, RALA is present at all points of the RALA + CD physical mixture. The change in a characteristic RALA peak in the red area of Figure 9 is shown in Figure 7, however, there are also district particles containing RALA in high concentration. Figure 9a showed remarkable differences between RALA + α-CD PM and SALA + α-CD PM, also between RALA + β-CD PM and SALA + β-CD PM. The red area in SALA + α-CD or β-CD PM was more observed than RALA + α-CD or β-CD PM. Ikeda *et al.* suggested that RALA bound more tightly to α-CD than SALA from the NMR study [23]. Kodama *et al.* showed that TM-β-CD worked as a chiral selector, which separating RALA and SALA in dietary supplement samples by capillary electrophoresis [31]. In this study, the same tendency could be observed that RALA might interact more tightly to α-CD than SALA in the physical mixtures.

Figure 9 proves that Raman mapping can detect the small amount of crystalline RALA which is below the limit of detection of the conventionally used XRD [22], even though the overall measurement time was approximately the same. In conclusion, complexation could take place for both RALA and SALA with all CDs. Despite a 1:1 molar ratio some of the active ingredient was detected in pure form. This could be because complexation was not complete or the complex is not thermodynamically stable and recrystallization occurs after the procedure.

These vibrational band changes confirm complex formation and offer an incremental information of complexation. It is quite difficult to explain these phenomena in the precise molecular level, however, at least we can say that the simple 1:1 complex of lipoic acid and CDs does not take place in every cases. It is true that further studies must be necessary involving some crystallographic analysis or computational works. In this meaning, our findings reported here will provide the milestone to make the progress in the host-guest chemistry.

## 3. Experimental Section

### 3.1. Chemicals

*R*(+)-α lipoic acid sodium salt (NaRALA) was purchased from ToyoHakko Co., Ltd. (Obu, Japan). *S*(–)-α lipoic acid sodium salt (NaSALA) was provided by Changshu Fushilai Medicine & Chemical Co., Ltd. (Changshu, China). CAVAMAX^®^ W6 FOOD (α-CD), CAVAMAX^®^ W7 FOOD (β-CD) and CAVAMAX^®^ W8 FOOD (γ-CD) were purchased from Wacker Chemie AG (Munich, Germany). The solvents used for spectrophotometry were purchased from Wako Pure Chemical Ind., Ltd. (Osaka, Japan). All reagents used were analytical grade and Milli Q^®^ water was used throughout the study. KBr was obtained from Merck (Merck, Darmstadt, Germany). The chemicals were used without further purification.

### 3.2. Equipments

The mechanical stirrer (EYELA NZ-1000) and the freeze dryer (EYELA FD-1000) used in this study were from Tokyo Rika Kikai, Tokyo, Japan. Differential scanning calorimetry (DSC) was carried out using the DSC-60 thermal analyzer (Shimadzu, Kyoto, Japan). The high pressure liquid chromatography (HPLC) system LC-2010 was equipped with a high-speed autosampler, a UV-Vis detector and LC solution^®^ chromatography data system software (Shimadzu). The scanning electron microscope (SEM) used was the S-4500 from Hitachi, Tokyo, Japan. The particle size distribution analysis was carried out using an LA-920 system (Horiba, Kyoto, Japan). Powder X-ray diffraction patterns (XRD) were measured with a Rint 2200 diffractometer (Rigaku, Tokyo, Japan).

### 3.3. Microscopic Fourier Transform Infrared Spectroscopy (Micro FT-IR)

The Nicolet iN 10 MX Infrared Imaging Microscope (Thermo Fisher Scientific, Waltham, MA, USA) was used. The powdered RALA-CD complexes were put on the clean glass plate and set on the equipped stage. The infrared light was focused on the test sample and the spectra were recorded in the range 4000–400 cm^−1^ in total reflection mode with a spectral resolution of 4 cm^−1^.

### 3.4. Microscopic-Raman Spectroscopy and Raman Chemical Imaging

A micro-Raman system (HORIBA Jobin Yvon, LabRAM HR800) equipped with a 633 nm He-Ne laser and an optical microscope (Olympus, BX41, Tokyo, Japan) was used. The laser beam was narrowed and focused through a 100 μm entrance pinhole and a 50× objective lens (Olympus, MPLN50X, NA = 0.75). Scattered light was collected in a backscattered geometry, in which an edge filter, a pinhole (1000 μm in diameter) and slit (100 μm in width) were positioned in front of the spectrometer.

Raman mapping spectra were collected using an external 514.5 nm Ar^+^ laser source. An objective of 10× magnification was used for optical imaging and spectrum acquisition. All spectra were obtained in the spectral range of 0–1300 cm^−1^ with approximately 1.5 cm^−1^ pixel resolution provided by a grating of 600 groove/mm and a 1024-pixels CCD detector which was attached to this micro-Raman system.

All samples were investigated in powder form. No further sample preparation was applied. Raman maps were collected with 10× objective (laser spot diameter: about three micron) and 40 μm step size. In each experiment the acquisition time of a single spectrum was 1 s and 10 spectra were added up at each spatial position. The measured area varied from 0.8 mm × 0.8 mm to 1 mm × 1 mm.

The reference Raman spectra of the pure ingredients were collected with a 50× objective using sufficient acquisition times to achieve adequate signal-to-noise ratio (the actual measurement time depended on the materials themselves).

## 4. Conclusions

The aim of this study was to understand host-guest interaction for RALA-CD and SALA-CD complexes and evaluate it using the micro-Fourier Transform Infrared Spectroscopy (micro-FT-IR) and micro-Raman spectroscopy. The micro-FT-IR analysis showed that the C=O stretching vibration of RALA appeared at 1717 cm^−1^ and shifted to 1708 (RALA-α-CD), 1733 (RALA-βCD), 1708 (RALA-γ-CD) cm^−1^ and the C=O stretching vibration of SALA appeared at 1717 cm^−1^ and shifted to 1709 (SALA-α-CD), 1735 (SALA-β-CD) and 1708 (SALA-γ-CD) cm^−1^, upon complexation with CDs. These results indicate that C=O of lipoic acid is located in the hydrophobic environment after forming complex with β-CD, whereas with α- or γ-CD, it is not. Micro-Raman spectroscopy showed that the S–S and C–S stretching vibrations, which appeared at 511 cm^−1^ (S–S), 631 cm^−1^ (C–S 2) and 675 cm^−1^ (C–S 1) for free RALA drastically weakened on formation of the RALA-CD complexes. The same results were obtained for SALA and its CD complexes. Raman Spectroscopic imaging indicated that complex formation could take place for both RALA and SALA with all CDs because the spectrum derived from free RALA and SALA was not observed for all RALA-CD and SALA-CD complexes. These results indicate that 1,2-dithiolane of ALA changes conformationally and the stretching vibration is inhibited after forming complexes with CDs. As for the chiral recognition of RALA and SALA is not still unclear, however, it is fair to say that the size recognition was not observed in these cases. In the case of the physical mixtures, the distributions of RALA and SALA were completely heterogeneous and they were present as distinct particles. For α-CD and β-CD, it could recognize the chirality of lipoic acid because the red area indicating lipoic acid was different between RALA and SALA physical mixtures (Figure 9a).

The shifts in the vibrational modes of FT-IR and Raman spectra certify the complex compound formation and offer an incremental information of the complexation process. Several peaks for O–H vibration shifted or the intensity changed. This indicates that CDs and RALA form host-guest complexes and that atomic interactions in the structure are large as causing significant changes in states of some chemical bonds. Further studies are needed to explore the O–H vibrations and the structural differences between RALA-CD and SALA-CD complexes in order to evaluate the chiral recognition of cyclodextrins.
